# An earthworm protease cleaving serum fibronectin and decreasing HBeAg in HepG2.2.15 cells

**DOI:** 10.1186/1471-2091-9-30

**Published:** 2008-11-24

**Authors:** Xue-Qing Wang, Lan Chen, Rong Pan, Jing Zhao, Ying Liu, Rong-Qiao He

**Affiliations:** 1 State Key Lab of Brain and Cognitive Sciences, Institute of Biophysics, the Chinese Academy of Sciences, 15 Da Tun Road, Chao Yang District, Beijing 100101, PR China; 2 School of Pharmaceutical Sciences, Peking University, 38 Xue Yuan Road, Hai Dian District, Beijing 100191, PR China; 3 Graduate University of Chinese Academy of Science, 19 A Yuquan Road, Shijingshan District, Beijing 100049, PR China

## Abstract

**Background:**

Virus-binding activity is one of the important functions of fibronectin (FN). It has been reported that a high concentration of FN in blood improves the transmission frequency of hepatitis viruses. Therefore, to investigate a protease that hydrolyzes FN rapidly is useful to decrease the FN concentration in blood and HBV infection. So far, however, no specific protease digesting FN in serum has been reported.

**Methods:**

We employed a purified earthworm protease to digest serum proteins. The rapidly cleaved protein (FN) was identified by MALDI-TOF MS and western blotting. The cleavage sites were determined by N-terminus amino acid residues sequencing. The protease was orally administrated to rats to investigate whether serum FN *in vivo *became decreased. The serum FN was determined by western blotting and ELISA. In cytological studies, the protease was added to the medium in the culture of HepG2.2.15 cells and then HBsAg and HBeAg were determined by ELISA.

**Results:**

The protease purified from earthworm *Eisenia fetida *was found to function as a fibronectinase (FNase). The cleavage sites on FN by the FNase were at R and K, exhibiting a trypsin alkaline serine-like function. The earthworm fibronectinase (EFNase) cleaved FN at four sites, R_259_, R_1005_, K_1557 _and R_2039_, among which the digested fragments at R_259_, K_1557 _and R_2039 _were related to the virus-binding activity as reported. The serum FN was significantly decreased when the earthworm fibronectinase was orally administrated to rats. The ELISA results showed that the secretion of HBeAg from HepG2.2.15 cells was significantly inhibited in the presence of the FNase.

**Conclusion:**

The earthworm fibronectinase (EFNase) cleaves FN much faster than the other proteins in serum, showing a potential to inhibit HBV infection through its suppressing the level of HBeAg. This suggests that EFNase is probably used as one of the candidates for the therapeutic agents to treat hepatitis virus infection.

## Background

Fibronectin (FN) is a glycoprotein composed of two nearly identical ~250 kDa subunits linked covalently near their C termini by a pair of disulfide bonds [1]. Each subunit is a mosaic of a series of repeating modules: 12 Type I, 2 Type II, 15 to 17 (depending on splicing) Type III modules, and a variable (V) sequence that is not homologous to other parts of FN. The 2 Type III modules called extradomain A (EDA) and extradomain B (EDB) are subject to alternative splicing [2]. On the basis of its solubility, FN is subdivided into two forms: soluble plasma FN and less-soluble cellular FN [1]. Plasma FN that is synthesized in hepatocytes contains neither EDA nor EDB whereas cellular FN (synthesized locally in tissues) contains variable amounts of either or both EDA and EDB. In plasma FN dimers, only one of the subunits contains the V region, whereas almost all cellular FN subunits contain this region [2]. Plasma FN is an abundant soluble constituent in plasma with a concentration about 300 μg/ml [1]; and cellular FN is an extracellular matrix that provides the architectural scaffolding in tissues [3].

FN is a multifunctional molecule involving many physiological and pathological processes such as infections with different viruses [4,5], cellular adhesion, embryogenesis, tissue damage and repair [6,7], atherosclerosis, myocardial infarction [2,8] and tumor therapy [9]. Studies have demonstrated that FN is involved in the virus infection, including rabies virus [10], human immunodeficiency virus (HIV) [11], cytomegalovirus [12], Rous sarcoma virus (RSV) [13] and infectious hematopoietic necrosis virus [4]. Recently, the interaction of hepatitis B virus (HBV) with FN has been investigated by several laboratories and their results have clarified that FN is related to HBV infection [14-18].

HBV infection is a worldwide public health problem, especially in the eastern countries. Globally, more than 350 million people are infected by HBV and 10~25% of them die of either liver cirrhosis or hepatocellular carcinoma [19]. Preventing the evolvement from transient HBV infection into chronic HBV, liver cirrhosis and hepatocellular carcinoma, human beings are encountering tremendous challenge. There are several therapeutic agents including interferon-α (IFN-α), peglyated IFN (PEG-IFN) and four oral nucleotide analogues (lamivudine, adefovir, entecavir and tonofovir) currently approved for the treatment of chronic HBV [20]. But there are no proteases as drugs in the therapeutic agents.

Previous studies have shown that FN is involved in HBV infection, liver cirrhosis and hepatocellular carcinoma. Budkowska and colleagues [14] have found that FN of human liver sinusoids is able to bind to HBV via the pre-s2 domain. This *in vivo *interaction of HBV with liver FN may play a role in the initial steps of virus's entry into the target cells, subsequently mediating by specific hepatocyte receptors. FN expression increases markedly in sinusoidal endothelial cells, and the matrixes deposit by these cells stimulate the conversion of liver lipocytes to myofibroblasts in rat hepatic fibrosis model. Matsui et al. [15] reported that cellular FN participates in the hepatic fibrogenesis and the hepatocellular carcinoma. In 2006, Wang and colleagues [16] used an antisense oligonucleotide (ASODN), fibronectin antibody, and a small molecular compound to treat HepG2.2.15 cell line, targeting to FN. Their results showed that a decrease in the concentration of FN has an anti-HBV activity in HepG2.2.15 cells.

Earthworms have long been used as materials to make drug for antipyretic and diuretic purposes in Chinese traditional medicine. Furthermore, some of earthworm proteases have been prepared into enteric-coated capsules for clinical use in China for more than 10 years [21,22]. Nakajima and co-workers [23] characterized six isozymes of serine proteases with fibrinolytic activity from the earthworm (*E. fetida *and *L. rubellus*). On the basis of their cleavage specificity against peptide substrates, some isozymes have both trypsin- and chymotrypsin-like activities, and some act in elastase-like function. Investigations of the enzymes are focused on cardiovascular system for a long time in this lab [24,25]. We found that the proteases can activate not only prothrombin but also plasminogen. However, whether the earthworm *E. fetida *synthesizes a protease to degrade FN rapidly has been still unknown.

In this study, we show a novel protease from *E. fetida*, which cleaves FN much more quickly than the other serum proteins *in vitro *and *in vivo*. Sequentially, the cleavage sites on FN by EFNase and the effect on the production of HBV antigens are also exhibited.

## Methods

### Materials

Human serum was got from health volunteers. FN (from human plasma, the homogeneity is evaluated by immunoelectrophoresis) and fibronectin antibody (developed in rabbit affinity isolated) were from Sigma-Aldrich (USA). Peroxidase-conjugated IgG was from Beijing Zhongshan Golden Bridge Biotechnology Co., LTD (China). Lamivudine was the product from Beijing Nordhuns Chemical Technology Co., LTD (China) and its purity was higher than 99% measured by HPLC. Sprague-Dawley (SD) rats were obtained from Vitalriver (China). The animal experiments adhered to the principles of care and use of laboratory animals and were approved by the Institutional Animal Care and Use Committee of Peking University Health Science Center.

### Cell culture

HepG2.2.15 cells were kindly provided by Dr Ding in the Beijing Institute of Radiation Medicine who got them from the Mount Sinai Medical Center in New York. Media for HepG2.2.15 cell culture consisted of Dulbecco's modified eagle medium (high glucose, Gibco, Invitrogen Corporation, USA) supplemented with 10% fetal bovine serum (PAA, Austria), antibiotic G-418 sulfate (final concentration 380 μg/ml, Amresco, USA) and penicillin-streptomycin solution (final concentrations 100 units/ml penicillin and 100 μg/ml streptomycin, HyClone, USA).

### Purification of EFNase

EFNase was purified by affinity chromatography as described [26]. Briefly, crude earthworm proteases were obtained from the ammonium sulfate precipitation of the soluble proteins of earthworm *E. fetida*. EFNase was purified on a 4-aminobenzamidine dihydrochloride-coupled Sepharose CL-6B affinity column (Pharmacia, USA) eluted with a gradient of denaturant from 0.1 to 1 M (AKTA prime plus, GE healthcare, Bio-science, USA). Eight amino acid residues of the N-terminus were sequenced (Applied Biosystem Automated Protein Sequencer, Applied Biosystem Inc., USA).

### Human serum fibronectin digested by EFNase

Human serum (25 μl) was incubated with different concentrations of EFNase (0, 0.4, 0.8, 1.6, 3.2, 6.4 and 10 μM) at 37°C. After 30 min incubation, aliquots were taken and diluted (10 times) immediately. The reaction was terminated by addition of 2 × sample buffer and boiling for 10 min. Samples (18 μl each) were electrophoresed on 8% SDS-PAGE. The protein bands in the gel were visualized with Coomassie Brilliant Blue R-250. Then, under the same conditions, the serum degradation in the presence of EFNase (final concentration 6.4 μM) was observed at different time intervals. Serum in the absence of EFNase and in the presence of trypsin (final concentration 6.4 μM) was used as negative and positive controls, respectively.

### Identification of 'fibronectin' in the serum using mass spectrometry

The quick-diminished protein band 'fibronectin' was digested by trypsin and subjected to MALDI-TOF MS [27]. The protein band of interest was excised from the gel and cut into small pieces. Another piece from a protein-free region of the gel was used as a parallel control for the digestion to identify trypsin autoproteolysis products. 25 mM NH_4_HCO_3 _in 50% acetonitrile was added to wash the gel particles and the gel particles dried in a vacuum centrifuge. 10 mM dithiothreitol in 25 mM NH_4_HCO_3 _was added, enough to cover the gel pieces and reduce at 56°C for 1 hr. Cooled to room temperature, the dithiothreitol solution was replaced roughly by the same volume 55 mM iodoacetamide in 25 mM NH_4_HCO_3_. Samples were incubated at room temperature in the dark with occasional vortexing for 45 min. The gel pieces were washed with 25 mM NH_4_HCO_3 _for 10 min while vortexing. They were dehydrated with 25 mM NH_4_HCO_3 _in 50% acetonitrile and rehydrated with 25 mM NH_4_HCO_3 _and dehydrated again. The liquid phase was removed and the gel pieces dried in a vacuum centrifuge. The gel particles were rehydrated in 25 mM NH_4_HCO_3 _(pH 8) containing 0.05–0.1 mg/ml trypsin by vortexing for 5 min. After incubated at 37°C for 12–16 h, the peptides were recovered from the gel particles and used for MALDI-TOF MS analysis (AXIMA-CFRTMPlus, Shimadzu, Japan). The peptide fingerprint was processed in the NCBI database using the MASCOT search program.

### Proof of the band 'fibronectin' by western blotting

Western blotting was performed to prove the character of the protein 'fibronectin' [28]. Different concentrations of EFNase were incubated with human serum at 37°C for 2 h or EFNase (final concentration 6.4 μM) was incubated with human serum at 37°C for different time intervals. Aliquots were taken for 8% SDS-PAGE. The proteins in the gel were electrotransferred onto nitrocellulose membrane (Millipore, USA). The membrane was blocked in phosphate buffer solution (PBS) containing 5.0% nonfat milk and then incubated with primary fibronectin antibody at 37°C for 2 h. After rinsing with PBS plus 0.2% Tween 20 (PBST), the membrane was subsequently exposed to the second antibody peroxidase-conjuated IgG in the same solution at 37°C for 2 h. Immunoreactive proteins were visualized by 3,3'-diaminobenzidine (DAB) staining, which generated a brown-colored product.

### Fibronectin concentration change after oral administration of EFNase

Six male rats (SD, weighing 200–250 g) were fasted overnight. Before oral administration of EFNase, blood samples were drawn from the ocular vein into tubes. Then EFNase was given by gavage. Blood samples were drawn at different time intervals (0.5, 1, 1.5, 2, 3, 4, 6, 8, and 26 h). Sera were prepared and stored at -20°C until determination. Western blotting of the sera was conducted as mentioned above to test the change of FN. The concentrations of FN in sera were determined by ELISA as described by Özkan et al [29-31] with some modification. Sera samples were diluted 1:50 and 50 μl aliquots were deposited on 96 well microtitration plates (Costar, USA). The plates were incubated at 37°C for 2 h followed by five washing with PBST buffer. Then the plates were blocked with 5% horse serum in PBS at 37°C for 2 h. The primary fibronectin antibody (diluted with PBS plus 5% horse serum at a ratio of 1:10,000) was added 50μl per well and the plates were incubated at 37°C for 2 h. The plates were then washed five times with PBST. Peroxidase-conjuated Ig G (diluted with PBS at a ratio 1:5000) was added 100 μl per well and the plates were incubated at 37°C for 2 h. After the plates were washed five times with PBST, substrate solution (3,3',5,5'-tetramethylbenzidine, TMB) 100 μl were dispensed per well with a multichannel pipet. The reaction was stopped by adding 50 μl/well 2.0 M sulfuric acid. After sufficient color development, absorbance values were determined with an ELISA reader at a wavelength of 450 nm immediately.

### Enzymolysis sites on fibronectin by EFNase

To investigate enzymolysis characteristics of EFNase, FN from human plasma was used. First, FN (1 mg/ml, 10 μl) incubated with EFNase at different final concentrations (0, 0.01, 0.03, 0.1, 0.3, and 3 μM, respectively) at 37°C for 15 min to determine the suitable concentration of EFNase to cleave FN. Then the proper concentration of EFNase (final concentration 0.3 μM) was incubated with FN for 1, 5, 10, 30 and 60 min, respectively. The cleaved products were electrophoresed on 8% reduced and non-reduced SDS-PAGE and electrotransferred onto polyvinylidene difluoride membrane (PVDF, Millipore, USA). After staining with Commassie Brilliant Blue R-250, the main digested fragments were cut out for amino acid sequencing to identify the main cleavage sites.

### Treatment of HepG2.2.15 cells by EFNase and lamivudine

HepG2.2.15 cells were seeded 1 × 10^4 ^cells per well in 96-well cell culture plates [16]. EFNase and lamivudine (an anti-HBV drug which is widely used in the therapy of chronic hepatitis B) were added to the media 3 days after seeding. Cells were grown in the presence of EFNase or lamivudine for 6 days with changes of medium every 3 days. Six days after treatment by EFNase or lamivudine. The culture supernatants were collected and subjected to HBsAg and HBeAg analysis.

### Assays of hepatitis B surface antigen and hepatitis B e antigen

Hepatitis B surface antigen (HBsAg) and hepatitis B e antigen (HBeAg) in culture media were determined using diagnostic kits for HBsAg and for HBeAg as described in the manufacturer's manual (Sino-American Biotechnology Co., China). The inhibition rates were calculated according to the following formula: inhibition rate (%) = (A_control _- A_test_)/A_control _× 100% [16].

### Cell viability assay

A colorimetric assay using CCK-8 was to assess cell viability [32]. After the cells in 96-well plates were treated with EFNase or lamivudine, the media were aspirated, and 10 μl of CCK-8 (Dojindo Labs, Japan) was added to each well and incubated at 37°C for 1 h. The absorbance at 450 nm that stands for cell growth was measured with the reference wavelength at 595 nm.

## Results

### Degradation of fibronectin in the presence of earthworm fibronectinase

**Figure 1:**
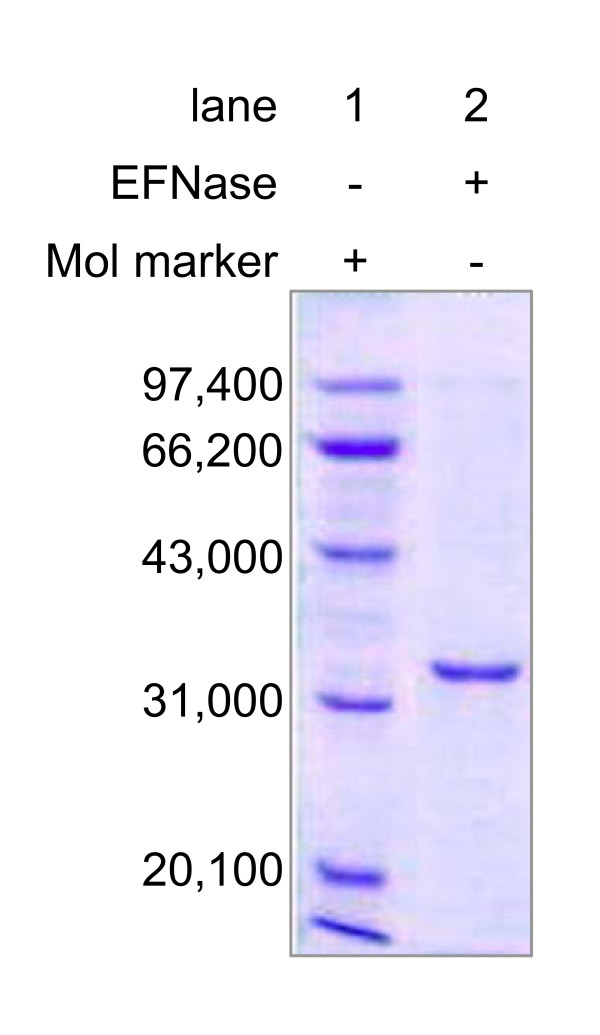
**Purification of EFNase**. SDS-PAGE of EFNase purified from *Eisenia fetida*. Lane 1 is the protein molecule markers; lane 2 represents EFNase.

The earthworm protease purified by ammonia sulfate precipitation followed by ionic and affinity chromatography columns showed a single band on SDS-PAGE with an apparent molecular mass of ~34 kDa (Figure 1). The N-terminal region was sequenced and the first eight amino acid residues were IVGGIEAR. The assay in the presence of Chromozym Try (a trypsin substrate) showed the specific activity of this protease was 176 units. One enzymic unit is defined as one mg of enzyme causing conversion of 1 μM of substrate per minute at 25°C.

**Figure 2:**
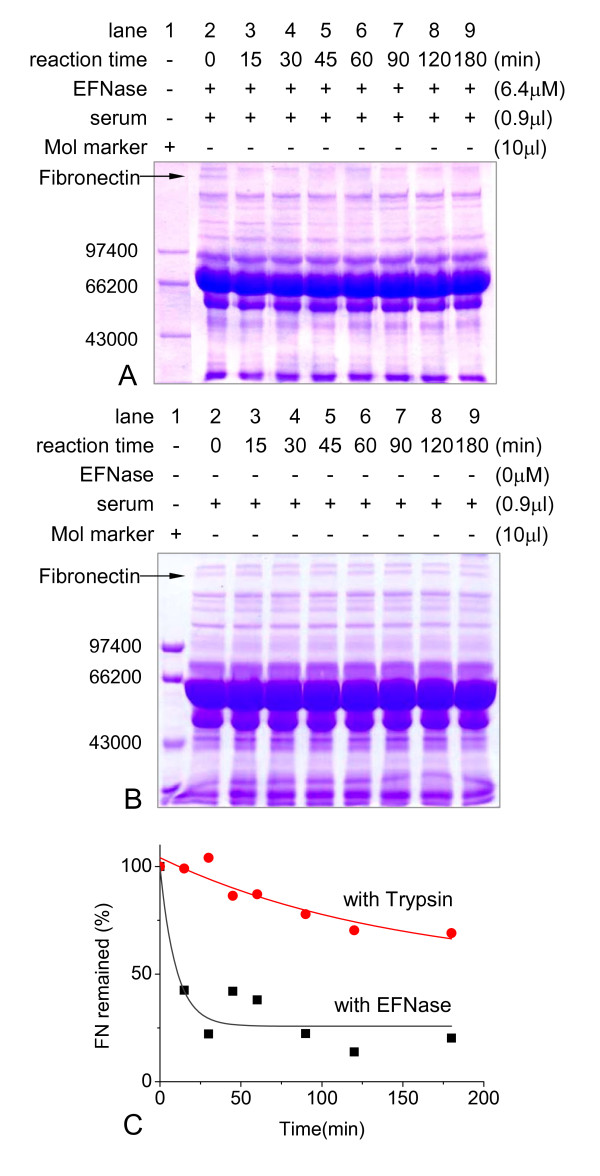
**Digestion of human serum in the presence of EFNase**. EFNase (final concentrations as indicated) was incubated with human serum (25 μl) at different time intervals during the degradation and then aliquots were taken for SDS-PAGE (panel A). Under the same conditions, serum in the absence of EFNase was used as negative control (panel B). Panel C was the comparison of the hydrolytic activity of EFNase and trypsin by densitomitric scanning analysis of Figure 2A and Additional file 1B.

SDS-PAGE of the human serum treated by different protease concentration was showed in Additional file 1A. Observably, a band of ~250 kDa protein in serum was rapidly degraded (at 37°C in 15 min) in the presence of the protease (final concentration 6.4 μM) (Figure 2A). Serum in the absence of the protease was used as a negative control, showing little changes in the protein bands in serum (Figure 2B). Trypsin (final concentration 6.4 μM) used as a positive control (Additional file 1B) depicted a relatively low hydrolytic activity on the ~250 kDa protein by the densitomitric scanning analysis (Figure 2C). This suggests that the earthworm protease has a significant high enzymic activity on the ~250 kDa protein.

**Figure 3:**
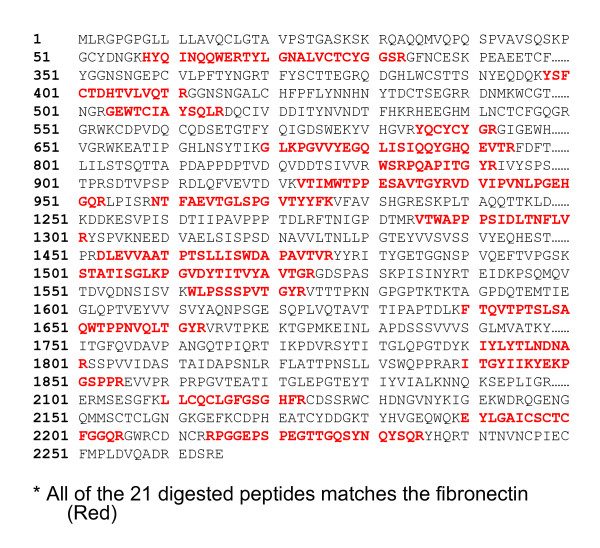
**The sequences of the matched peptides**. Match to fibronectin 1, Homo sapiens, Q60FE4, GenBank.

Now, the ~250 kDa protein should be identified. Mass spectrometry was first employed (Additional file 2). Analysis on MALDI-TOF MS showed a peptide fingerprint of 'fibronectin' with degradation by trypsin. The database searching results indicated that the protein was 'fibronectin' with a molecular mass of ~252,842 Da. Among 31 peptides, 21 matched to the theoretical protonated molecular masses of FN tryptic fragments within 50 ppm (Additional file 3). The sequences of the matched peptides (match to Q60FE4 human, FN1, Homo sapiens) were showed in Figure 3.

**Figure 4:**
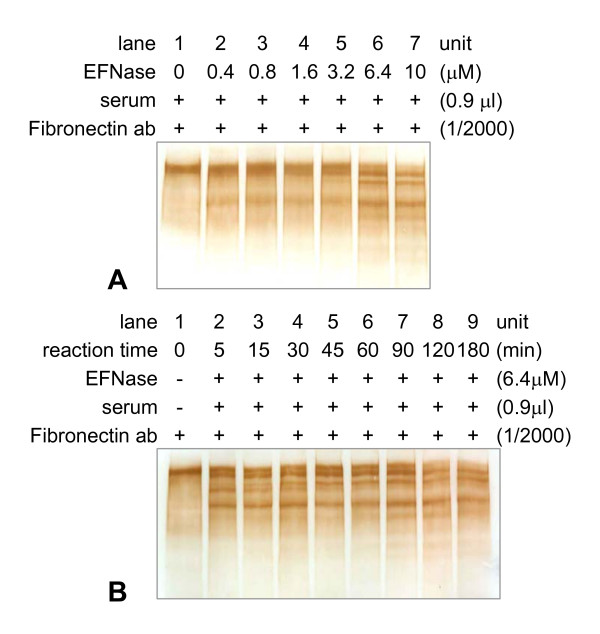
**The further proof of 'fibronectin' in serum by western blotting**. EFNase (final concentrations as indicated) was incubated with human serum (25 μl) at 37°C for 120 min, and then aliquots were taken for SDS-PAGE and western blotting (panel A). EFNase was also added to serum and aliquots were taken for SDS-PAGE and western blotting at different time intervals during the degradation (panel B).

To further prove the band of 'fibronectin', western blotting was employed. The fibronectin antibody was able to recognize the serum protein well. When different concentrations of the earthworm protease (from 0.4 to 10 μM) were incubated with human serum at 37°C for 2 h (Figure 4A), the ~250 kDa serum protein was rapidly degraded. Furthermore, in the presence of the protease (6.4 μM, 37°C), the serum protein was markedly digested in 5 min (Figure 4B). The digestion also showed concentration- and time-dependent character.

**Figure 5:**
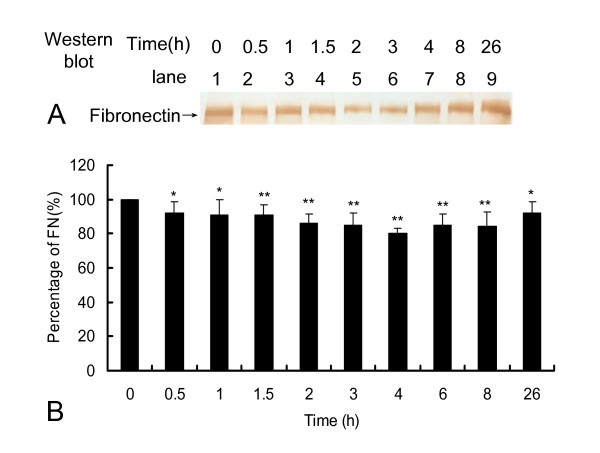
**Changes in fibronectin concentrations after EFNase oral administrated to rats**. EFNase was oral administrated to rats at a dose 2000 mg/kg. Panel A was a typical western blotting picture of the serum. Lane 1 showed the serum before oral administration of EFNase as control; Lanes 2 through 9 were serum after oral administration of EFNase at time as indicated. Panel B was the FN concentrations determined by ELISA (Mean ± SD, n = 6). * *P *< 0.05, **P < 0.01 *versus *the FN concentration before oral administration of EFNase.

Sequentially, the effect of EFNase on FN *in vivo *should be investigated. As shown in Figure 5A, a typical western blot pictured the degradation of FN in the rat serum after oral administration of EFNase. The gray density of FN band was descent and then ascent. The relative content of serum FN significantly decreased from 1 to 4 h. ELISA results showed that the mean lowest FN concentration in blood occurred at ~4 h (Figure 5B). Therefore, the serum protein is fibronectin (FN) and thus the protease is called earthworm fibronectinase (EFNase).

### Cleavage sites on fibronectin

**Figure 6:**
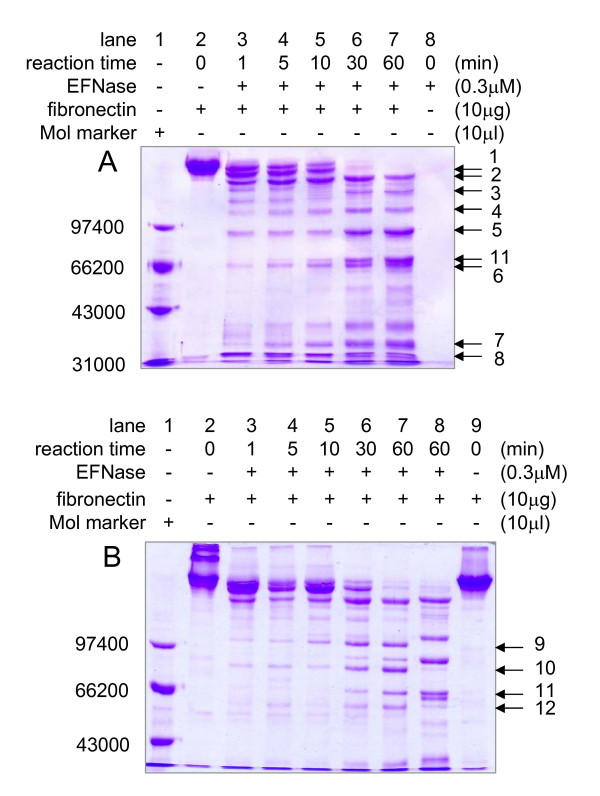
**Digestion of fibronectin by EFNase**. EFNase (final concentrations as indicated) was incubated with FN at 37°C and aliquots were taken at different time intervals for reducing SDS-PAGE during the degradation (panel A). Under the same conditions, FN in the presence of EFNase was analyzed by non-reducing SDS-PAGE, compared with reducing SDS-PAGE. Lanes 2 through 7 were non-reducing SDS-PAGE and lanes 8 to 9 were reducing SDS-PAGE (panel B).

The digested FN fragments released from the incubation with EFNase were shown in Figure 6. Human plasma fibronectin existed mainly as 250 kDa monomer on the reduced SDS-PAGE (Additional file 4A, lane 6). As the enzyme concentration increased, the ~250 kDa monomer was degraded, resulting in some smaller polypeptides (Additional file 4A, lanes 1–5) and exhibiting a concentration-dependent character. Almost none of intact FN remained when EFNase concentration was 3 μM. Thus, 0.3 μM of EFNase was employed to carry out the time-dependent experiment (Figure 6A). On the gel, FN monomer disappeared within 1 min followed by appearance of digested bands. On reducing (Figure 6A) and Non-reducing (Figure 6B) SDS-PAGE, over ten digested fragments appeared. With reference to the complete primary structure of human FN (NP002017, GenBank, mature peptide part), four cleavage sites were determined (Table 1). Seven fragments (bands 1, 2, 3, 4, 5, 9 and 10) cleaved at R_259_/A_260 _(I), two (bands 6 and 12) at K_1557_/T_1558 _(III), one (band 7) at R_2039_/H_2040 _(IV) and one (band 11) at R_1005_/G_1006 _(II) were exhibited. Band 8 represented an N-terminal region fragment.

**Table 1:** Cleavage sites on fibronectin by EFNase

Fragment	No. cleavage site	Cleavage site
1,2,3,4,5,9,10	I	R_259_/A_260_
6,12	III	K_1557_/T_1558_
7	IV	R_2039_/H_2040_
8		from Q_1 _to R_259_
11	II	R_1005_/G_1006_

The amino acid sequence of FN is referred to fibronectin 1 isoform 3 preproprotein [NP002017, GenBank, mature peptide part].

**Figure 7:**
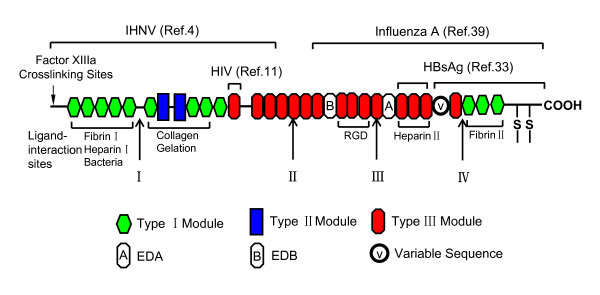
**The main function domain of fibronectin digested by EFNase**. The functional domains of FN digested by EFNase were shown in this panel. Ligands interaction regions and cleavage sites are shown (discussed in text). Variably spliced extradomain A (A) and extradomain B (B) modules and variable sequence (V) are shown in the subunit of the FN dimer.

The schematic diagram of the modular structure of FN monomer is showed in Figure 7. The four cleavage sites are also indicated on the diagram. From N-terminal to C-terminal, the first cleavage site on FN is R_259_/A_260_(I). This domain contains the N-terminal region that is involved in crosslinking to coagulation factor XIIIa. Five Type I repeats are also in this region, which is bound to fibrin, heparin and bacteria. From Figure 6A, bands 1, 2 and 8 appear quickly, showing the sensitive site of R_259_/A_260 _to EFNase. According to the N-terminal amino acid sequence (QAQQMVQPQ) and the apparent molecular mass (about 30 kDa), we deduced band 8 is the fragment from Q_1 _to R_259_. The second cleavage site is at R_1005_/G_1006 _(II). The collagen and gelation binding domain is reported to localize between R_259_/A_260 _and R_1005_/G_1006 _[1]. The third cleavage site is at K_1557_/T_1558_(III). The important integrin-recognition motif RGD (in repeat III_10_) is located in this part. The strongest heparin-binding site is between the third and the fourth cleavage site. A binding site for HBsAg is from the variable sequence (V) to C-terminus [33]. The fourth cleavage site (R_2039_/H_2040_) is located at this region.

**Figure 8:**
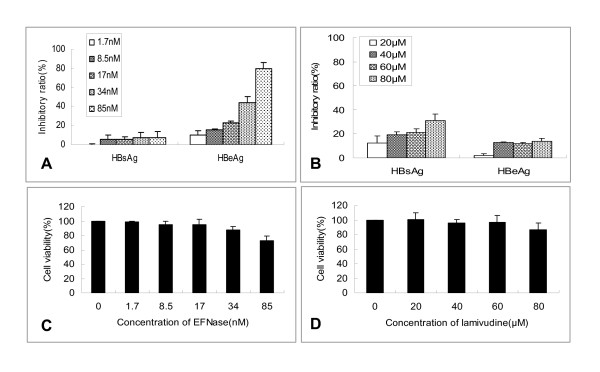
**Effect of EFNase on HBeAg and HBsAg secreted HepG2.2.15 cells and the cell viability**. HBsAg and HBeAg in cell culture in the presence of EFNase (panel A) and lamivudine (panel B) were analyzed by ELISA. Cell viabilities treated with EFNase (panel C) and lamivudine (panel D) were determined using CCK-8 kits. The data represent means ± SD (n = 3).

The main cleavage sites were at R and K, indicating a trypsin alkaline serine-like protease of EFNase. BSA was used as a control for the degradation, but the EFNase (0.3 μM) depicted a little hydrolysis on BSA (Additional file 4B). This suggests again that EFNase has a relatively high specificity to FN.

### HBsAg and HBeAg assays after the cells treated by EFNase and lamivudine

Because fibronectinolysis may disturb the virus binding activity to FN, EFNase was used to treat HepG2.2.15 cells to test its potential function in HBV infection. HepG2.2.15 cells were the clonal cells derived from HepG2 cells, transfected with a plasmid containing HBV DNA. The cells secreted not only all HBV proteins but also hepatitis B virions, whilst they were remarkably down-regulated by lamivudine [34]. As shown in Figure 8A and 8B, EFNase and lamivudine were observed to inhibit the secretion of HBsAg and HBeAg in HepG2.2.15 cell culture medium. The ELISA results indicated that the secretion of HBeAg was decreased by EFNase much more obvious than by lamivudine. However, the inhibition of HBsAg secretion in the presence of EFNase was less than lamivudine under the experimental conditions.

To assess the potential toxicity of EFNase and lamivudine to HepG2.2.15 cells, the viability of treated cells was examined. The results showed that cell viability had little change when the concentration of EFNase was lower than 34 nM under the experimental conditions (Figure 8C). Lamivudine had little impact on the cell growth when its concentration reached 100 μM (Figure 8D).

## Discussion

In this study, the earthworm FNase has been found to recognize the ~250 kDa protein from human serum and digest it rapidly. We think that this serum protein is FN because of the following results. (1) The molecular mass of the protein is ~250 kDa, approximately equal to FN (from 210 to 250 kDa). (2) The N-terminal amino acid sequence is identical to that of FN (QAQQMVQPQ). (3) The mass-spectrum shows a fingerprint of FN for the trypsin digested serum protein. Finally, (4) EFNase degrades the purified FN at the specific cleavage sites as mentioned above.

It is necessary to explain why we call the earthworm protease as EFNase. First, we have known that R and K are the main cleavage sites for the protease, exhibiting the trypsin alkaline serine-like activity. 0.01 μM of the protease digests FN within 15 min, showing a high hydrolytic activity to FN. Definitely, the protease is able to recognize FN from a great number of proteins contained in human serum. Under the same conditions, trypsin as a control did not rapidly degrade the serum FN. The earthworm protease did not quickly digest BSA and the other serum proteins. By *in vivo *experiments we have demonstrated that the relative concentration of the serum FN will decrease when the earthworm protease are orally administrated to rats. These results have given us an enlightenment to consider the protease as an FNase.

As shown in the previous studies, the earthworm *E. fetida *contains 8 trypsin-like proteases and their molecular masses are around 30 kDa [35]. The apparent molecular mass of the earthworm FNase is ~34 kDa (Figure 1), similar to these proteases. The N-terminal sequence (8 amino acid residues) of EFNase showed identical to that of one isozymes of the earthworm protease (*Ef*P-III-2, a trypsin-like protease). However, the complete gene and the intact amino acid sequence of EFNase have not been clarified. Whether EFNase is *Ef*P-III-2 remains further investigated.

To study the cleavage site of EFNase on FN, we have employed plasma FN as the substrate. Although plasma FN is distinct from cellular FN in some functions [36,37], most of the amino acid sequence is similar between plasma FN and cellular FN except EDA, EDB and V regions. Plasma FN is much more soluble than the tissue FN. Thus, it is convenient for us to analyze the degraded fragments of plasma FN in the presence of EFNase. From the results of plasma FN, we could predict some changes of cellular FN in the presence of the earthworm proteases. In fact, our unpublished data show that the concentration of cellular FN in the HBV transgenic mice livers is indeed decreased.

One of the important functions of FN is virus-binding activity. A high concentration of FN in blood improves the transmission frequency of hepatitis viruses [38]. Several domains have been reported to participate in the viruses binding to FN (see Figure 7). Liu and co-workers [4] have shown that FN2 (with a sequence from N-terminus to Type III_3_) mediates IHNV attachment and cell entry. HIV-1_IIIB _specifically interacts with the III_1_-C region within FN and the interaction may play a role in facilitating HIV infection in vivo [11]. The C-terminal 120–140 kDa fragment that carries the cell-binding activity bound influenza A (H3N2, Bangkok strain) virus most efficiently [39]. A binding site for HBsAg has been found in heparin binding region II, and between the variable sequence (V) and C-terminus [33]. This virus-binding ability may explain the high rate of transmission of virus infections by plasma derivates enriched in FN [38]. In this study, we hypothesized that the cleavage of FN by EFNase may have the possibility to influence the binding activity of viruses to FN and virus infections. However, the FN fragments may elicit quite different function from the intact molecule [40]. The cleavage products increase or decrease the binding effect needs further investigation.

For the cytological experiments, HBsAg and HBeAg secreted by HepG2.2.15 cells were both inhibited when the cells were treated with EFNase, especially the HBeAg decreases more obviously. HBeAg has the best correlation to the presence of infectious HBV. It is an important marker of the infection. Once HBeAg seroconversion takes place, there may be transition to the nonreplicative phase, characterized by normal serum liver enzymes and low HBV DNA levels and reduced hepatic necroinflammation [41]. The significant clearance of HBeAg by EFNase may endow EFNase a potential product to treat HBV infection diseases.

The mechanism for EFNase to decrease HBeAg level in the HepG2.2.15 cell culture is due to two possibilities. (1) The cleavage of FN by EFNase may disturb the virus-binding activity and may inhibit the initial steps of the virus entry into target cells. (2) EFNase degrades HBeAg directly to decrease the level of the antigen in the medium. But, this possibility should not exist because HBsAg would be digested if the EFNase substrate specificity was so broad that all the proteins in the medium should be destroyed. In general, the mechanism for EFNase to decreases HBeAg remains further to be studied.

We have tried to find a drug with the same mechanism as a positive control to the inhibition of the antigens preduced by HepG2.2.15 cells. But no drug with such a characteristic was found. Lamivudine is selected because it is the first-line drug in treating chronic HBV infections on clinic [20]. In research on HBV infections, lamivudine is commonly used as a positive control. Meanwhile, lamivudine, an oral antiviral agent, has the same administration route with EFNase.

We adopted an oral administration route of the protease to develop an oral administration dosage form. The FN concentration in serum decreased when EFNase was orally administrated to rats. As we know, earthworm proteases have been formulated into the oral enteric-soluble capsules which protect the enzymes from degradation in stomach. This formulation whose efficiency is certified has been successfully used on clinic for more than ten years [21,22]. Furthermore, previous [42-44] and our present (Additional file 5) investigations on the absorption of the proteases indicate that the proteases could be partially absorbed from intestines to blood.

As mentioned above, the enzyme has a high activity to hydrolyze FN in serum. However, the protease could hydrolyze some other proteins under the experimental conditions (Figure 2). Furthermore, this protease is supposed to digest some other proteins on the HepG 2.2.15 cells or in the serum of rats although we didn't detect the degradation of the proteins.

An important cleavage site should be emphasized here is R_1005_/G_1006_, which is involved in the binding of collagen (Type I_6_–I_9 _and II_1_–II_2_). Cutting at this site may destroy the interaction between FN and collagen. In fact, some HBV infection evolves into liver cirrhosis and hepatocellular carcinoma. FN is important in the fibrogenesis. During the early phase of active fibroplasia, FN production increases dramatically, and this augmentation is associated with the fibroblast proliferation thereafter responsible for excessive synthesis and deposition of the collagen protein. FN associated with collagen through the specific binding sites, which promotes the formation of fibre. The hydrolysis of FN by EFNase not only decreases FN concentration directly, but also prevents the association of collagen to FN, which probably delay the formation of fibre.

The current therapeutic agents (including interferon and nucleoside analogues) used for the treatment of chronic HBV are mainly prevent the replicate of the viruses. But once the cirrhosis has occurred, it is difficult to be reversed [45]. EFNase is supposed to be able to prevent both the processes, which has some advantage over interferon and nucleoside analogues. Thus we think that EFNase may have the potential to be developed into a new medicine to treat chronic HBV, liver cirrhosis and hepatocellular carcinoma patients.

## Conclusion

In conclusion, a novel EFNase that cleaved FN much faster than the other proteins in mammal serum was purified from *E. fetida*. The results suggest that EFNase may have the potential to inhibit HBV infection through its suppressing the level of FN and HBeAg.

## Competing interests

The authors declare that they have no competing interests.

## Authors' contributions

All authors contributed to the conception and design of the article, along with the acquisition and interpretation of the results. All authors were involved in revising the article for important intellectual content and approved the final version to be published.

## Supplementary Material

Additional file 1 **Digestion of human serum in the presence of EFNase.** EFNase (final concentrations as indicated) was incubated with human serum (25 μl) at 37°C for 30 min, and then aliquots were taken for SDS-PAGE (panel A). Serum in the present of trypsin was used as positive control (panel B). Click here for file

Additional file 2 **The identification of 'fibronectin' in the serum using mass spectrometry.** The peptide fingerprint of 'fibronectin' was analyzed by MALDI-TOF MS after digested by trypsin. Click here for file

Additional file 3 **The mass/charge and sequences of matched peptides in the determination of 'fibronectin' in the serum using mass spectrometry.** Click here for file

Additional file 4 **Digestion of fibronectin by EFNase.** EFNase (final concentrations as indicated) was incubated with FN at 37°C for 15 min, and then aliquots were taken for reducing SDS-PAGE (panel A). BSA in the presence of EFNase was used as a control (panel B). Click here for file

Additional file 5 **Immunoblotting of EFNase in the medium of serosal side during mucosal-to-serosal transport and immunohistochemistry analysis of the intestinal epithelium.** The everted sac model for studying intestinal transport of large peptides and proteins has been used here. Mucosal-to-serosal transport (duodenum segment of small intestine) of EFNase was performed. Aliquots (5 μL) of serosal medium were taken at different incubation time intervals for immunoblotting as indicated (panel A). Lane 6 indicated the full-sized EFNase as a positive control. The gray shade densities of immunoreactive bands were shown on panel B. Incubated with EFNase (final concentration 10 μM) at the mucosal side for 30 min, the everted intestinal segment was sectioned and immunologically visualized in the presence of anti-EFNase serum as primary antibody (panel C). Those in the absence of the primary antibody were used as controls (panel D). The results indicated that the intact EFNase could be transported from the mucosal to serosal side. Click here for file
