# Supplementary material for: An earthworm protease cleaving serum fibronectin and decreasing HBeAg in HepG2.2.15 cells
Source: BMC Biochem. 2008 Nov 24;9:30. doi: 10.1186/1471-2091-9-30 (PMC2611985; doi:10.1186/1471-2091-9-30)

lane	1	2	3	4	5	6	7	8	
EFNase	0.4	0.8	1.6	3.2	6.4	10	-	-	(μM)
serum	+	+	+	+	+	+	+	-	(0.9μl)
Mol marker	-	-	-	-	-	-	-	+	(10μl)

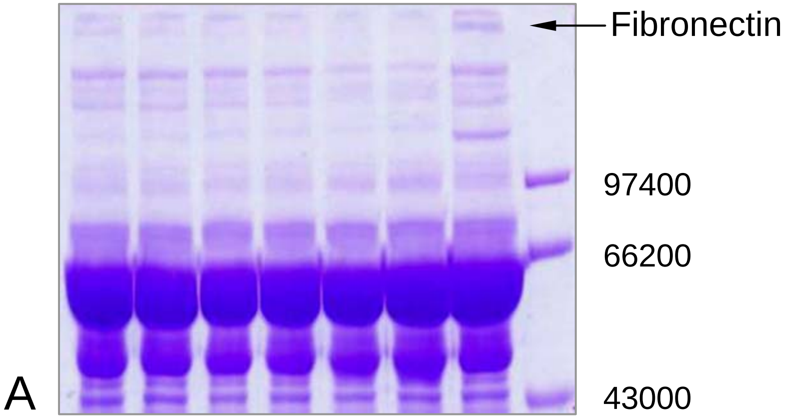

lane	1	2	3	4	5	6	7	8	9	
reaction time	15	30	45	60	90	120	180	0	-	(min)
Trypsin	+	+	+	+	+	+	+	-	-	(6.4μM)
serum	+	+	+	+	+	+	+	+	-	(0.9μl)
Mol marker	-	-	-	-	-	-	-	-	+	(10μl)

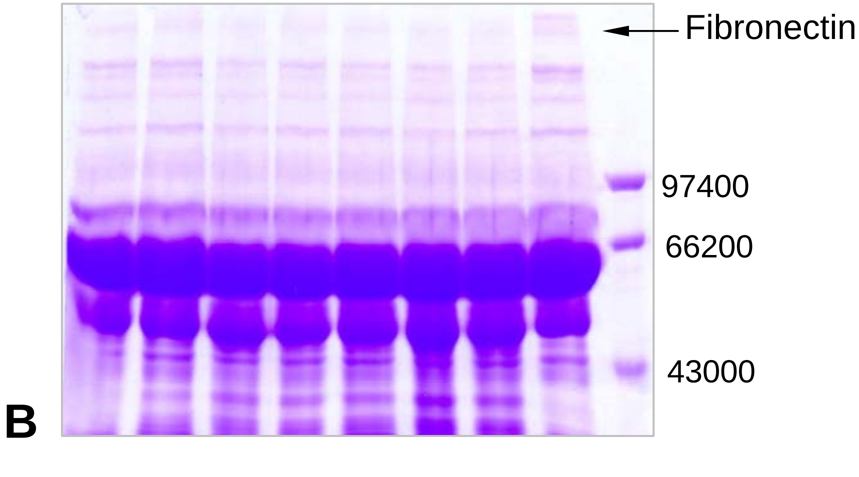

Supplement: Additional file 1 — Digestion of human serum in the presence of EFNase. EFNase (final concentrations as indicated) was incubated with human serum (25 μl) at 37°C for 30 min, and then aliquots were taken for SDS-PAGE (panel A). Serum in the present of trypsin was used as positive control (panel B). [file 1471-2091-9-30-S1.pdf]
