# Supplementary figures and images for: An earthworm protease cleaving serum fibronectin and decreasing HBeAg in HepG2.2.15 cells
Source: BMC Biochem. 2008 Nov 24;9:30. doi: 10.1186/1471-2091-9-30 (PMC2611985; doi:10.1186/1471-2091-9-30)

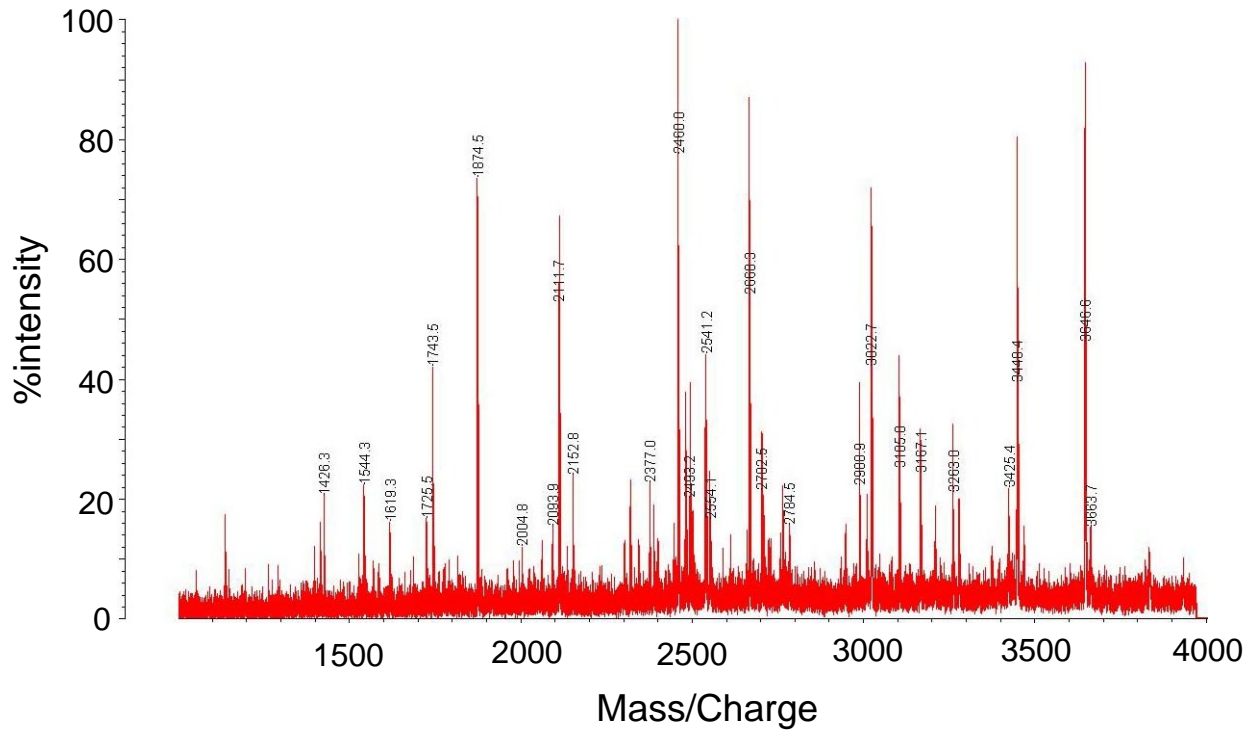

Supplement: Additional file 2 — The identification of 'fibronectin' in the serum using mass spectrometry. The peptide fingerprint of 'fibronectin' was analyzed by MALDI-TOF MS after digested by trypsin. [file 1471-2091-9-30-S2.pdf]

**A**

|           |   |    |    |    |     |   |
|-----------|---|----|----|----|-----|---|
| lane      | 1 | 2  | 3  | 4  | 5   | 6 |
| Aliquot   | + | +  | +  | +  | +   | - |
| EFNase    | - | -  | -  | -  | -   | + |
| Time(min) | 5 | 15 | 30 | 60 | 120 | - |

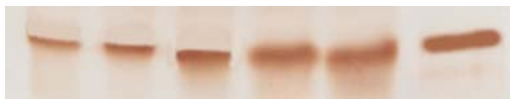**B**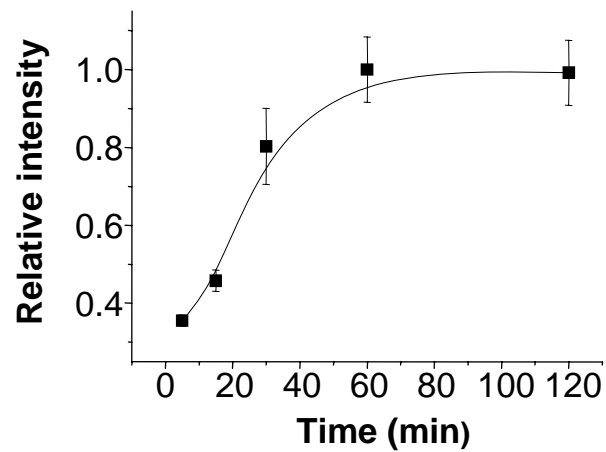**C**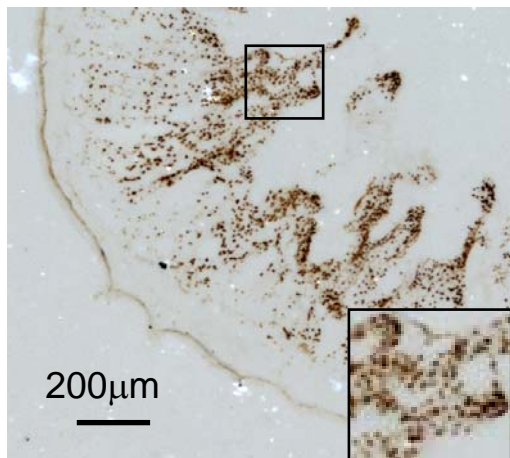**D**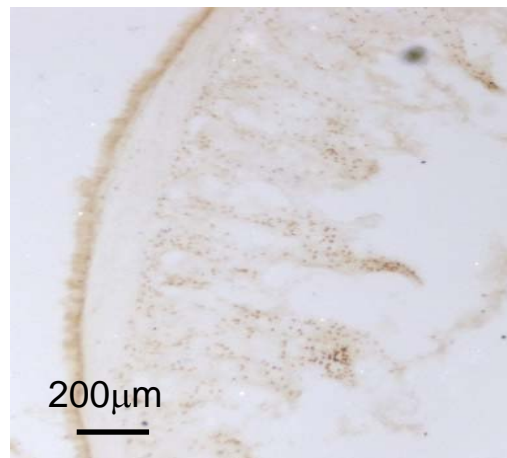

Supplement: Additional file 5 — Immunoblotting of EFNase in the medium of serosal side during mucosal-to-serosal transport and immunohistochemistry analysis of the intestinal epithelium. The everted sac model for studying intestinal transport of large peptides and proteins has been used here. Mucosal-to-serosal transport (duodenum segment of small intestine) of EFNase was performed. Aliquots (5 μL) of serosal medium were taken at different incubation time intervals for immunoblotting as indicated (panel A). Lane 6 indicated the full-sized EFNase as a positive control. The gray shade densities of immunoreactive bands were shown on panel B. Incubated with EFNase (final concentration 10 μM) at the mucosal side for 30 min, the everted intestinal segment was sectioned and immunologically visualized in the presence of anti-EFNase serum as primary antibody (panel C). Those in the absence of the primary antibody were used as controls (panel D). The results indicated that the intact EFNase could be transported from the mucosal to serosal side. [file 1471-2091-9-30-S5.pdf]
