# Supplementary material for: An earthworm protease cleaving serum fibronectin and decreasing HBeAg in HepG2.2.15 cells
Source: BMC Biochem. 2008 Nov 24;9:30. doi: 10.1186/1471-2091-9-30 (PMC2611985; doi:10.1186/1471-2091-9-30)
Supplement: Additional file 3 — The mass/charge and sequences of matched peptides in the determination of 'fibronectin' in the serum using mass spectrometry. [file 1471-2091-9-30-S3.doc]

## Additional file 3 - The Mass/Charge and Sequences of matched peptides in the determination of ‘fibronectin’ in the serum using mass spectrometry.

| Peptide (Start-End) | Mass/Charge（Observed） | Sequence of matched peptides |
| --- | --- | --- |
| 58 - 67 | 1401.7700 | K.HYQINQQWER.T |
| 68 - 83 | 1791.9300 | R.TYLGNALVCTCYGGSR.G |
| 398 - 411 | 1726.9000 | K.YSFCTDHTVLVQTR.G |
| 504 - 515 | 1483.8100 | R.GEWTCIAYSQLR.D |
| 585 - 592 | 1169.5200 | R.YQCYCYGR.G |
| 670 - 694 | 2799.5100 | K.GLKPGVVYEGQLISIQQYGHQEVTR.F |
| 831 - 842 | 1431.8400 | R.WSRPQAPITGYR.I |
| 923 - 938 | 1807.9800 | K.VTIMWTPPESAVTGYR.V |
| 923 - 938 | 1823.9900 | K.VTIMWTPPESAVTGYR.V Oxidation (M) |
| 939 - 953 | 1629.9700 | R.VDVIPVNLPGEHGQR.L |
| 959 - 976 | 1994.1000 | R.NTFAEVTGLSPGVTYYFK.V |
| 1285 - 1301 | 1926.1500 | R.VTWAPPPSIDLTNFLVR.Y |
| 1453 - 1476 | 2524.4700 | R.DLEVVAATPTSLLISWDAPAVTVR.Y |
| 1501 - 1524 | 2470.4900 | K.STATISGLKPGVDYTITVYAVTGR.G |
| 1562 - 1573 | 1349.7900 | K.WLPSSSPVTGYR.V |
| 1640 - 1663 | 2692.5100 | K.FTQVTPTSLSAQWTPPNVQLTGYR.V |
| 1791 - 1801 | 1355.7900 | K.IYLYTLNDNAR.S |
| 1840 - 1855 | 1819.0400 | R.ITGYIIKYEKPGSPPR.E |
| 2110 - 2123 | 1651.9300 | K.LLCQCLGFGSGHFR.C |
| 2190 - 2205 | 1878.9100 | K.EYLGAICSCTCFGGQR.G |
| 2214 - 2235 | 2396.2400 | R.RPGGEPSPEGTTGQSYNQYSQR.Y |
