# Supplementary material for: An earthworm protease cleaving serum fibronectin and decreasing HBeAg in HepG2.2.15 cells
Source: BMC Biochem. 2008 Nov 24;9:30. doi: 10.1186/1471-2091-9-30 (PMC2611985; doi:10.1186/1471-2091-9-30)

lane	1	2	3	4	5	6	7	
EFNase	.03	.01	.3	.1	3	0	-	(μM)
fibronectin	+	+	+	+	+	+	-	(10μg)
Mol marker	-	-	-	-	-	-	+	(10μl)

A

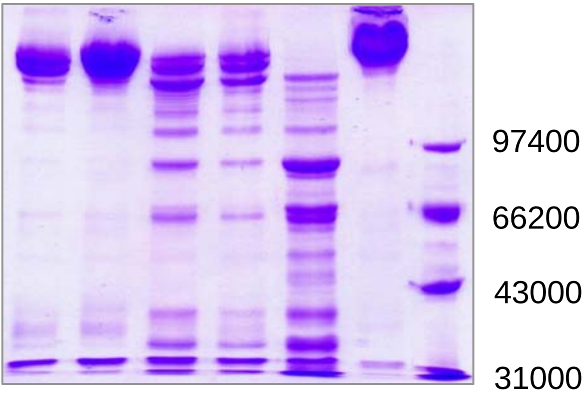

lane	1	2	3	4	5	6	7	8	
reaction time	-	0	1	5	10	30	60	0	(min)
EFNase	-	-	+	+	+	+	+	+	(0.3μM)
BSA	-	+	+	+	+	+	+	-	(5μg)
Mol marker	+	-	-	-	-	-	-	-	(10μl)

B

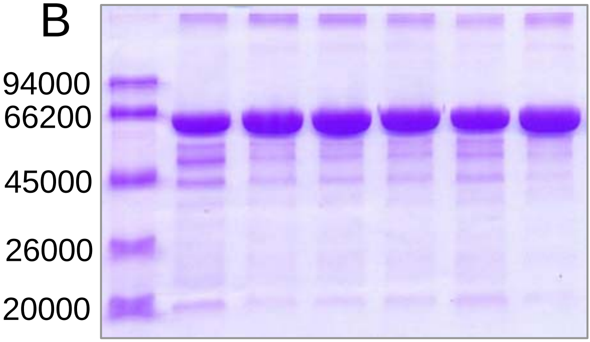

Supplement: Additional file 4 — Digestion of fibronectin by EFNase. EFNase (final concentrations as indicated) was incubated with FN at 37°C for 15 min, and then aliquots were taken for reducing SDS-PAGE (panel A). BSA in the presence of EFNase was used as a control (panel B). [file 1471-2091-9-30-S4.pdf]
